# Supplementary material for: Weather anomalies more important than climate means in driving insect phenology
Source: Commun Biol. 2023 May 5;6:490. doi: 10.1038/s42003-023-04873-4 (PMC10163234; doi:10.1038/s42003-023-04873-4)
Supplement: Supplementary file 2 — Supplemental Material [file 42003_2023_4873_MOESM2_ESM.pdf]

Supplemental Table 1. Model parameters for onset, offset and duration models for models fit using GDD. For onset models, we used 60 prior days of unusual warm and cold; for offset, these were measured between onset and offset, and for duration, the sum of the first two. Below we simply refer to "unusual cold days" and "unusual warm days" to simplify presentation of results. Parameters with model coefficients whose 95% Bayesian credible interval does not include zero are bolded. If a model parameter was dropped in the final model or not included, we left parameter estimate value blank.

Predictors	Onset	Offset	Duration
Intercept	**122.6 (109.0 - 136.5)**	**228.8 (212.8 - 244.7)**	**104.6 (89.4 - 119.7)**
Accumulated GDD	**-10.6 (-13.0 - -7.6)**	**-7.0 (-8.8 - -5.1)**	**9.2 (6.1 - 12.3)**
Precipitation		**1.8 (0.2 - 3.5)**	2.1 (-0.2 - 4.2)
Unusual cold days	**-4.8 (-6.4 - -3.2)**	**8.3 (5.3 - 11.2)**	**13.7 (10.5 - 17.0)**
Unusual warm days	**-2.3 (-3.7 - -0.8)**	**3.2 (0.9 - 5.5)**	**2.8 (1.0 - 4.6)**
Voltinism [Uni]	**-8.1 (-14.2 - -2.2)**	**-47.6 (-55.8 - -39.6)**	**-36.0 (-43.6 - -28.4)**
Overwintering strategy [Egg]	**48.9 (31.2 - 66.1)**	17.2 (-3.3 - 37.7)	**-29.9 (-49.2 - -10.5)**
Overwintering strategy [Larvae]	**29.6 (14.7 - 44.1)**	-0.5 (-17.6 - 16.8)	**-30.7 (-46.8 - -14.4)**
Overwintering strategy [Migratory]	**24.8 (4.2 - 45.3)**	15.4 (-11.2 - 41.2)	-7.5 (-31.5 - 16.5)
Overwintering strategy [Pupae]	**22.7 (7.1 - 38.1)**	**-9.1 (-27.6 - 9.7)**	**-30.4 (-47.6 - -13.0)**
GDD:Unusual cold days		**1.8 (0.3 - 3.4)**	
GDD:Voltinism [Uni]			**-6.5 (-10.3 - -2.6)**
Unusual cold days:Unusual warm days	**-2.1 (-3.7 - -0.5)**		
Unusual warm days:Precipitation		**2.6 (0.7 - 4.5)**	**4.0 (2.0 - 6.0)**
Unusual cold days:Voltinism [Uni]		**-5.4 (-9.2 - -1.5)**	**-6.3 (-10.4 - -2.2)**
Unusual warm days:Voltinism [Uni]		**-3.6 (6.9 - -0.3)**	
Distinct observation days		**5.0 (3.3 - 6.7)**	**6.6 (4.6 - 8.6)**
Conditional R2	0.8	0.87	0.84

Supplemental Table 2. Table of species and their traits used in this analysis. Abbreviations for overwinteringStage: E=egg, L=larval, P=pupal. Abbreviations for voltinism: U=univoltine, M=multivoltine.

scientificName	overwinteringStage	voltinism	Seasonality
Achalarus lyciades	L	M	Summer
Acronicta hasta	P	M	Summer
Acronicta impressa	P	M	Summer
Acronicta interrupta	P	M	Summer
Aglais milberti	A	M	Spring
Aglossa cuprina	L	U	Summer
Agonopterix pulvipennella	A	U	Spring
Amphipyra pyramidoides	E	U	Summer
Anagrapha falcifera	P	M	Summer
Ancyloxypha numitor	L	M	Summer
Anthocharis midea	P	U	Spring
Apamea sordens	L	U	Summer
Apantesis phalerata	L	M	Summer
Asterocampa clyton	L	M	Summer
Autographa precationis	L	M	Summer
Biston betularia	P	M	Summer
Boloria bellona	L	M	Summer
Boloria frigga	L	U	Summer
Callophrys augustinus	P	U	Spring
Callophrys niphon	P	U	Spring
Campaea perlata	L	M	Summer
Carterocephalus palaemon	L	U	Summer
Catocala amatrix	E	U	Fall
Catocala amica	E	U	Fall
Catocala andromedae	E	U	Summer
Catocala antinympha	E	U	Summer
Catocala cara	E	U	Fall
Catocala coccinata	E	U	Fall
Catocala concumbens	E	U	Fall
Catocala crataegi	E	U	Summer
Catocala grynea	E	U	Fall
Catocala habilis	E	U	Fall
Catocala ilia	E	U	Summer
Catocala innubens	E	U	Fall
Catocala lineella	E	U	Fall
Catocala micronympha	E	U	Summer
Catocala mira	E	U	Summer
Catocala neogama	E	U	Fall
Catocala obscura	E	U	Fall
Catocala palaeogama	E	U	Fall
Catocala parta	E	U	Fall
Catocala praeclara	E	U	Summer
Catocala relicta	E	U	Fall
Catocala retecta	E	U	Fall
Catocala similis	E	U	Summer
Catocala ultronia	E	U	Fall

Celastrina ladon	P	M	Spring
Celastrina serotina	P	U	Spring
Cercyonis pegala	L	U	Summer
Chlosyne harrisii	L	U	Summer
Cisseps fulvicollis	P	M	Summer
Cisthene packardii	L	M	Summer
Coenonympha tullia	L	M	Summer
Colias eurytheme	P	M	Summer
Colias philodice	L	M	Summer
Colocasia propinquilinea	P	M	Summer
Costaconvexa centrostrigaria	A	M	Summer
Cyaniris neglecta	P	M	Summer
Danaus plexippus	M	M	Summer
Darapsa myron	P	M	Summer
Desmia funeralis	P	M	Summer
Ectropis crepuscularia	P	M	Summer
Ennomos magnaria	E	U	Fall
Epargyreus clarus	P	M	Summer
Epimecis hortaria	L	M	Spring
Erynnis baptisiae	L	M	Summer
Erynnis brizo	L	U	Summer
Erynnis icelus	L	M	Summer
Erynnis juvenalis	L	U	Spring
Estigmene acrea	L	M	Summer
Euphydryas phaeton	L	U	Summer
Euphyes conspicua	L	U	Summer
Euphyes vestris	L	M	Summer
Euptoieta claudia	A	M	Summer
Fabricius dorcas	E	U	Summer
Glaucopsyche lygdamus	P	U	Spring
Halysidota tessellaris	P	M	Summer
Harkenclenus titus	E	U	Summer
Hesperia leonardus	L	U	Fall
Hesperia sassacus	L	U	Summer
Heterocampa guttivitta	P	U	Summer
Idia americalis	L	M	Summer
Junonia coenia	M	M	Summer
Lacanobia subjuncta	P	M	Summer
Lethe appalachia	L	M	Summer
Limenitis archippus	L	M	Summer
Limenitis arthemis	L	M	Summer
Lon hobomok	L	U	Summer
Lon zabulon	L	M	Summer
Lophocampa caryae	P	U	Summer
Lycaena phlaeas	M	M	Summer
Lymantria dispar	E	U	Summer
Malacosoma americanum	E	U	Summer

Metanema inatomaria	P	U	Spring
Nadata gibbosa	P	M	Summer
Noctua pronuba	M	M	Summer
Nymphalis antiopa	A	U	Spring
Oeneis polixenes	L	U	Summer
Orthosia hibisci	P	U	Spring
Panopoda rufimargo	P	U	Summer
Panthea acronyctoides	P	U	Summer
Panthea furcilla	P	M	Summer
Pantographa limata	L	U	Summer
Paonias excaecatus	P	M	Summer
Paonias myops	E	M	Summer
Papilio canadensis	P	U	Summer
Papilio glaucus	P	M	Summer
Papilio troilus	P	M	Summer
Pholisora catullus	L	M	Summer
Phragmatobia fuliginosa	L	M	Summer
Phyciodes tharos	L	M	Summer
Pieris rapae	P	M	Summer
Pieris virginiensis	P	U	Spring
Poanes viator	L	M	Fall
Polites mystic	L	U	Summer
Polites origenes	L	U	Summer
Polites peckius	P	M	Summer
Polites themistocles	P	M	Summer
Polygonia comma	A	M	Spring
Polygonia interrogationis	A	M	Summer
Pompeius verna	L	M	Summer
Satyrium acadica	E	M	Summer
Satyrium calanus	E	U	Summer
Satyrium caryaevorus	E	U	Summer
Satyrium liparops	E	U	Summer
Smerinthus cerisyi	P	U	Summer
Speranza pustularia	E	U	Summer
Speyeria aphrodite	L	U	Summer
Speyeria cybele	L	U	Summer
Spilosoma virginica	L	M	Summer
Strymon melinus	P	M	Summer
Thorybes pylades	L	M	Summer
Thymelicus lineola	E	U	Summer
Tolype laricis	E	U	Fall
Vanessa atalanta	A	M	Summer
Vanessa cardui	M	M	Summer
Vanessa virginiensis	A	M	Summer
Wallengrenia egeremet	L	M	Summer
Xestia c-nigrum	L	M	Summer

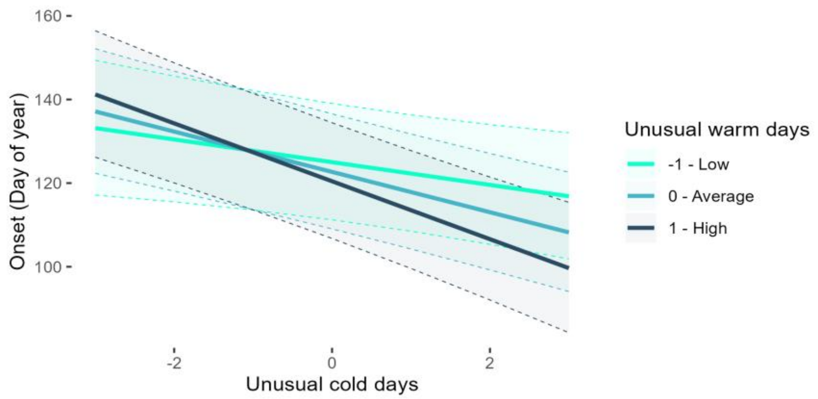

Supplemental Figure 1. Effect plots showing flight start dynamics in relation to number of unusual warm days and cold days. Onset of adult flight period is earliest when there are unusual numbers of warm and cold days.

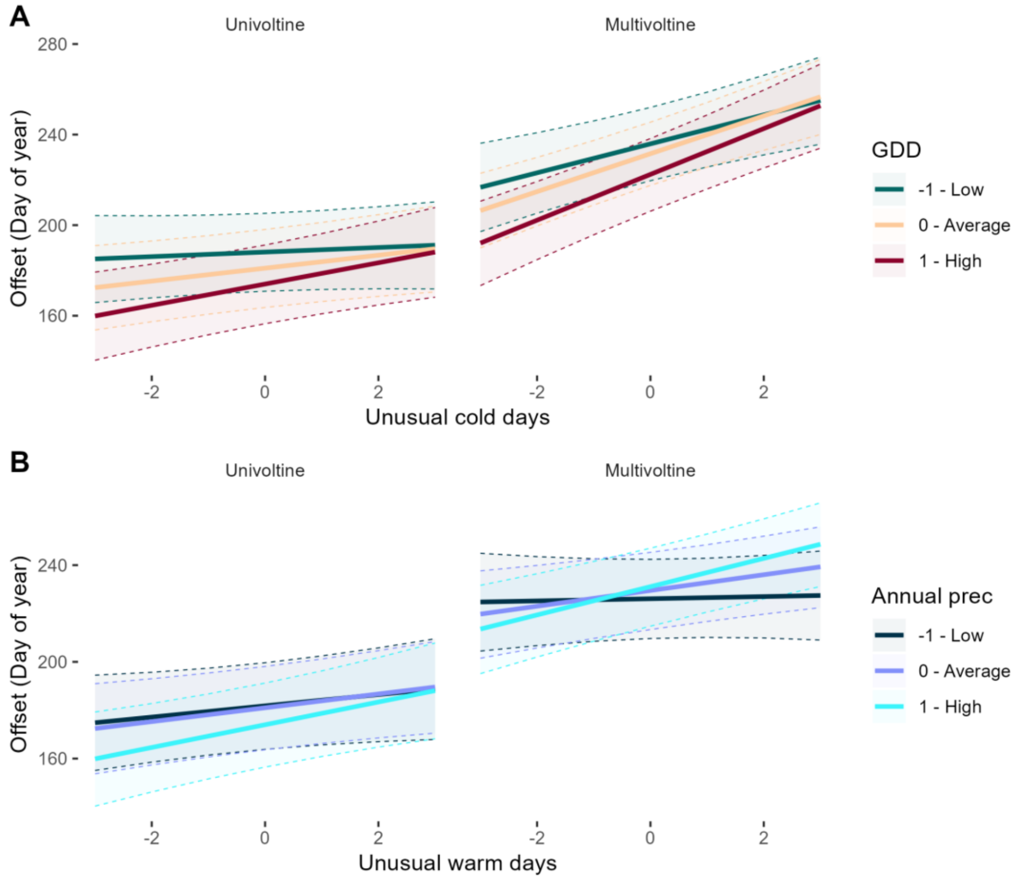

Supplemental Figure 2. Effect plots showing termination dynamics for uni- and multivoltine species in relation to number of unusual cold and warm days and GDD. **Panel A**: Multivoltine species' flight termination timing is more sensitive to unusual cold days than univoltine, but both show longer durations with more unusual cold and are more sensitive to unusual cold days in areas with high GDDs. **Panel B**. Offset is later in higher precipitation regimes for both uni- and mulivoltine species.

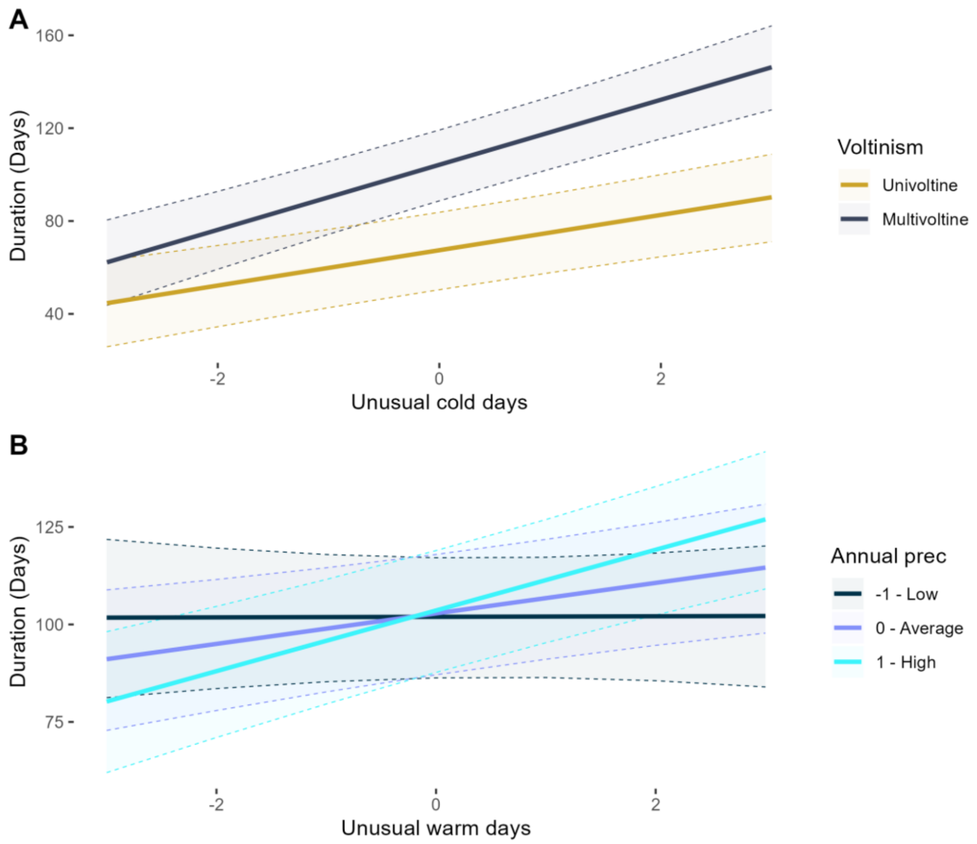

Supplemental Figure 3. Effect plots showing duration of flight in relation to number of unusual cold days and voltinism, showing stronger sensitivity for multivoltine species. Higher annual precipitation leads to shorter duration when there are fewer unusually warm days and longer durations when there are more unusually warm days.

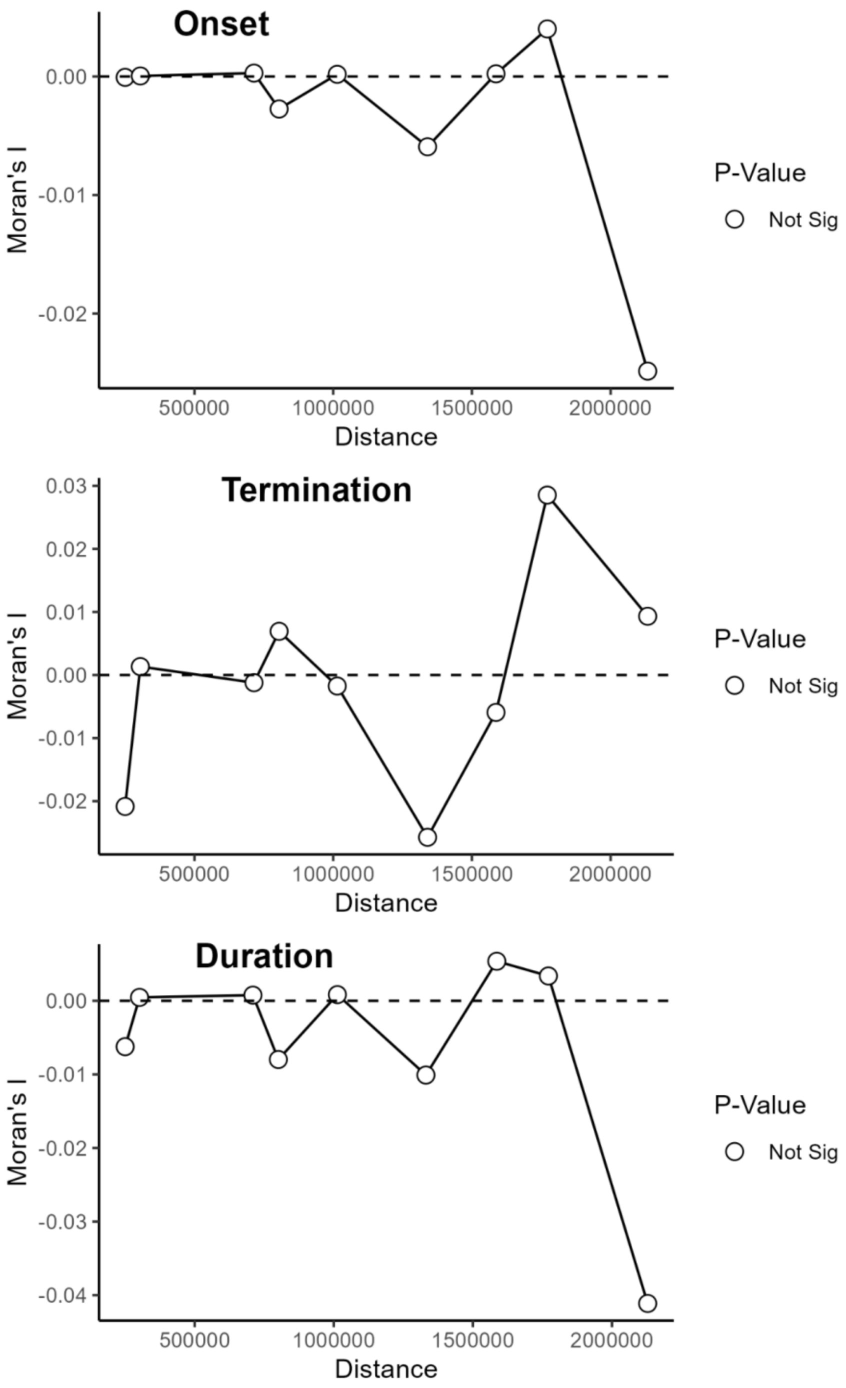

Supplemental Figure 4. AFC plots showing a bar chart of correlation coefficients between years and year lags based on residuals from onset, offset and duration PGLMM models for GDD models. While we do find some evidence of 15 year lag effects for onset and offset, these are not strong effects and may be within the range of expected false positives for multiple comparisons.

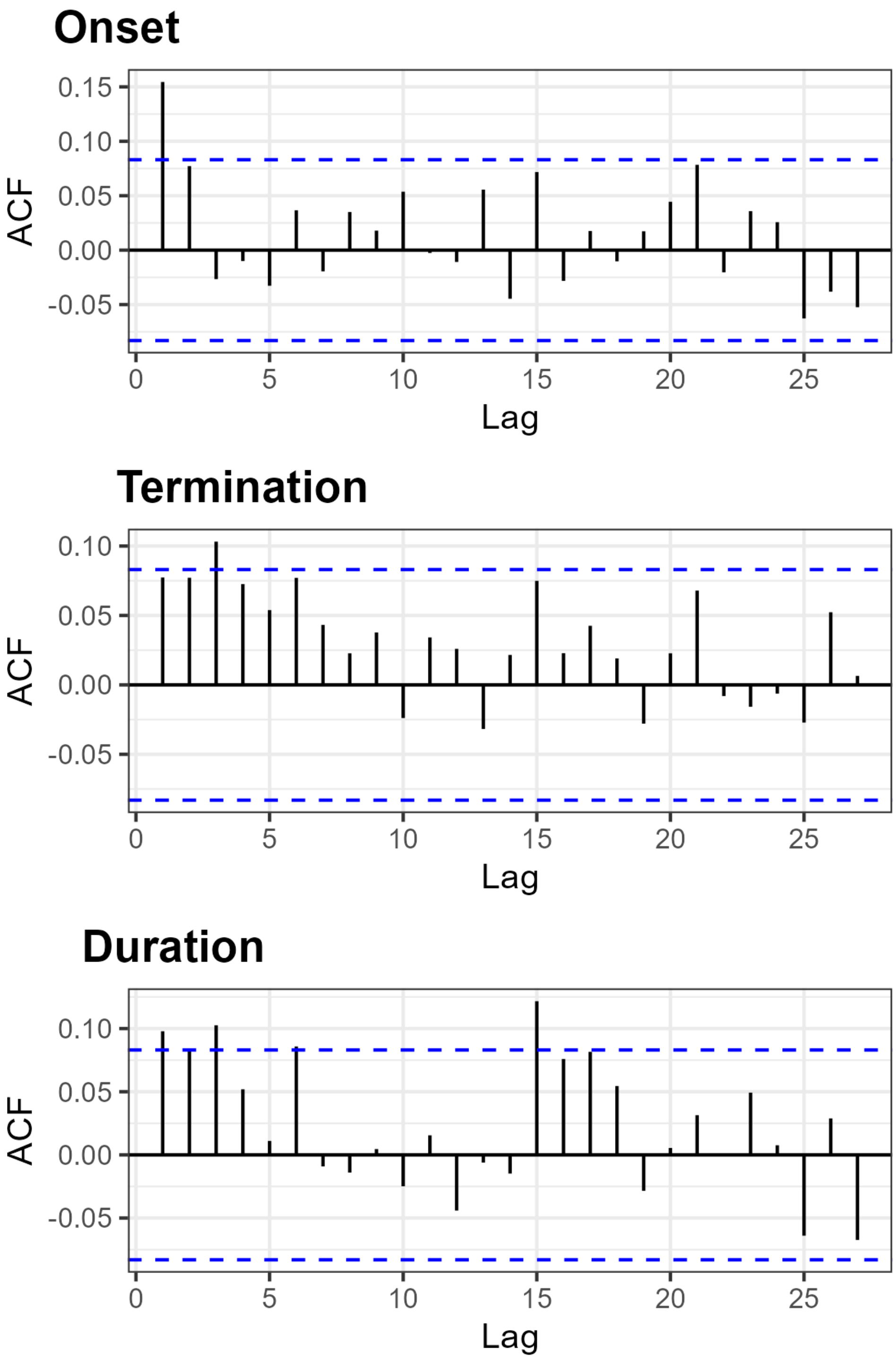

Supplemental Figure 5. AFC plots showing a bar chart of correlation coefficients between years and year lags based on residuals from onset, offset and duration PGLMM models. While we do find some evidence of 15 year lag effects for onset and offset, and weak early year lag effects for duration and termination, these are not strong effects and may be within the range of expected false positives for multiple comparisons.

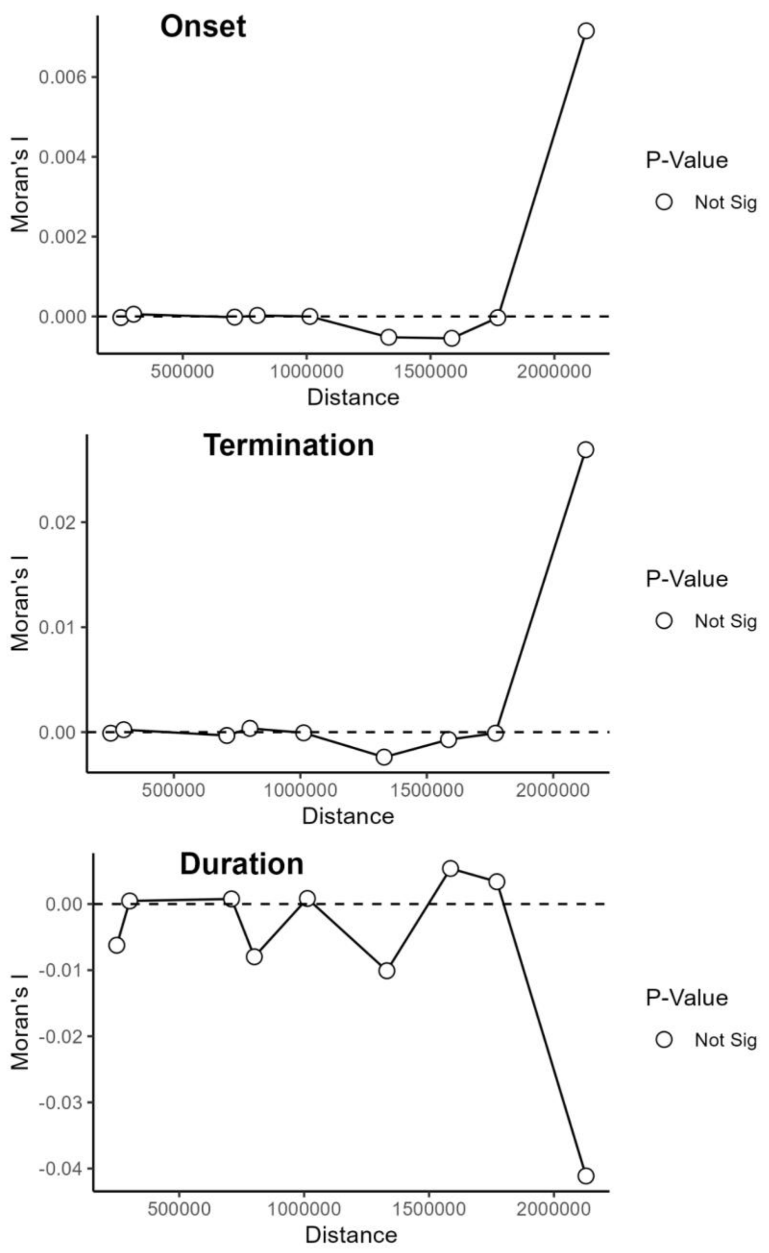

Supplemental Figure 6. Spatial correlogram showing Moran's I versus distance for onset, offset and duration annual temperature models described in the main text. This plot suggests no clear evidence of spatial autocorrelation across any phenology stage, except at the most distant spatial lags.

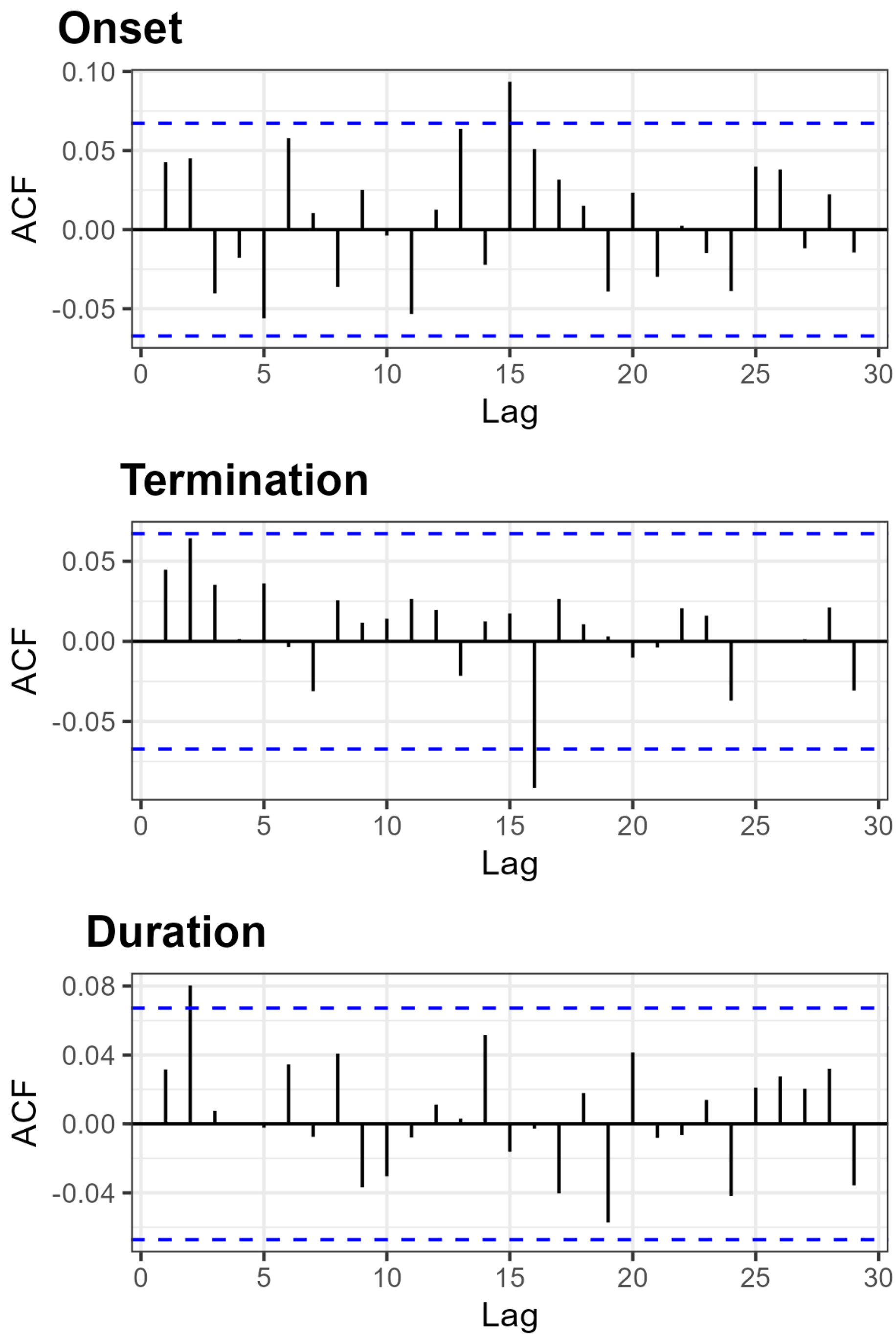

Supplemental Figure 7. AFC plots showing a bar chart of correlation coefficients between years and year lags based on residuals from onset, offset and duration PGLMM annual temperature models described in the main text. While we do find some evidence of 15 year lag effects for onset and offset, and 2 year lag effects for duration, these are not strong effects and may be within the range of expected false positives for multiple comparisons.
