# Supplementary material for: Weather anomalies more important than climate means in driving insect phenology
Source: Commun Biol. 2023 May 5;6:490. doi: 10.1038/s42003-023-04873-4 (PMC10163234; doi:10.1038/s42003-023-04873-4)
Supplement: Supplementary file 3 — Reporting Summary [file 42003_2023_4873_MOESM3_ESM.pdf]

nature portfolio

Corresponding author(s): Guralnick

Last updated by author(s): 4/21/2023

Reporting Summary

Nature Portfolio wishes to improve the reproducibility of the work that we publish. This form provides structure for consistency and transparency in reporting. For further information on Nature Portfolio policies, see our Editorial Policies and the Editorial Policy Checklist.

Statistics

For all statistical analyses, confirm that the following items are present in the figure legend, table legend, main text, or Methods section.

n/a	Confirmed	
☒	☐	The exact sample size (*n*) for each experimental group/condition, given as a discrete number and unit of measurement
☒	☐	A statement on whether measurements were taken from distinct samples or whether the same sample was measured repeatedly
☒	☐	The statistical test(s) used AND whether they are one- or two-sided<br>*Only common tests should be described solely by name; describe more complex techniques in the Methods section.*
☐	☒	A description of all covariates tested
☐	☒	A description of any assumptions or corrections, such as tests of normality and adjustment for multiple comparisons
☐	☒	A full description of the statistical parameters including central tendency (e.g. means) or other basic estimates (e.g. regression coefficient) AND variation (e.g. standard deviation) or associated estimates of uncertainty (e.g. confidence intervals)
☒	☐	For null hypothesis testing, the test statistic (e.g. *F*, *t*, *r*) with confidence intervals, effect sizes, degrees of freedom and *P* value noted<br>*Give P values as exact values whenever suitable.*
☒	☐	For Bayesian analysis, information on the choice of priors and Markov chain Monte Carlo settings
☐	☒	For hierarchical and complex designs, identification of the appropriate level for tests and full reporting of outcomes
☐	☒	Estimates of effect sizes (e.g. Cohen's *d*, Pearson's *r*), indicating how they were calculated

Our web collection on statistics for biologists contains articles on many of the points above.

Software and code

Policy information about availability of computer code

Data collection	We used custom R code for assembling and filtering datasets as described in the Methods and Data and Code Availability section
Data analysis	We used R for all analyses and figure generation as described in the Methods and in the Data and Code Availability section

For manuscripts utilizing custom algorithms or software that are central to the research but not yet described in published literature, software must be made available to editors and reviewers. We strongly encourage code deposition in a community repository (e.g. GitHub). See the Nature Portfolio guidelines for submitting code & software for further information.

Data

Policy information about availability of data

All manuscripts must include a data availability statement. This statement should provide the following information, where applicable:

- Accession codes, unique identifiers, or web links for publicly available datasets
- A description of any restrictions on data availability
- For clinical datasets or third party data, please ensure that the statement adheres to our policy

The data (phenoestimates, traits, climate data etc.) can be found on GitHub (https://github.com/robgur/LepPheno_UnusualWeather), which is a fork of the Github repository https://github.com/mbelitz/LepPheno_BestPractices, since phenoestimates used here came from that other work. Raw occurrence records needed to replicate our workflow can be downloaded and unzipped from our Open Science Framework project (https://osf.io/wdzay/).

Research involving human participants, their data, or biological material

Policy information about studies with human participants or human data. See also policy information about sex, gender (identity/presentation), and sexual orientation and race, ethnicity and racism.

Reporting on sex and gender	NA
Reporting on race, ethnicity, or other socially relevant groupings	NA
Population characteristics	NA
Recruitment	NA
Ethics oversight	NA

Note that full information on the approval of the study protocol must also be provided in the manuscript.

Field-specific reporting

Please select the one below that is the best fit for your research. If you are not sure, read the appropriate sections before making your selection.

☐ Life sciences ☐ Behavioural & social sciences ☒ Ecological, evolutionary & environmental sciences

For a reference copy of the document with all sections, see nature.com/documents/nr-reporting-summary-flat.pdf

Ecological, evolutionary & environmental sciences study design

All studies must disclose on these points even when the disclosure is negative.

Study description	We calculated phenometrics derived from insect natural history collections data, and used linear mixed models with key climatic covariates and species as random effects in order to test the hypothesis that unusually warm or cold days impact onset, offset and duration of adult butterfly and moth flight.
Research sample	We used a set of moth and butterfly natural history collections data for species found in eastern North America. These data included location, date and taxon, and we aggregated these records into a 250X250km grid cells.
Sampling strategy	We used a set of filters to remove species where key sample size criteria were not met, and we also filtered out outliers based on best practices that we have developed.
Data collection	We used digitized natural history collections data available from iDigBio and GBIF. These were cleaned using a best practices pipeline.
Timing and spatial scale	We used data from 1948-2016 and delimited our spatial scope to Eastern North America.
Data exclusions	We excluded species, cells and years where phenoestimates were prone to very high uncertainty based on best practices and simulations we had previously performed.
Reproducibility	All raw data, assembled phenometrics, traits, climate data and code to re-run analyses are available as per the data and code availability statements.
Randomization	We are not concerned with randomization in this design.
Blinding	We are not concerned with blinding in this design.

Did the study involve field work? ☐ Yes ☒ No

Reporting for specific materials, systems and methods

We require information from authors about some types of materials, experimental systems and methods used in many studies. Here, indicate whether each material, system or method listed is relevant to your study. If you are not sure if a list item applies to your research, read the appropriate section before selecting a response.

Materials & experimental systems

n/a	Involved in the study
☒	☐ Antibodies
☒	☐ Eukaryotic cell lines
☒	☐ Palaeontology and archaeology
☒	☐ Animals and other organisms
☒	☐ Clinical data
☒	☐ Dual use research of concern
☒	☐ Plants

Methods

n/a	Involved in the study
☒	☐ ChIP-seq
☒	☐ Flow cytometry
☒	☐ MRI-based neuroimaging
